# Supplementary material for: Contributions of cell behavior to geometric order in embryonic cartilage
Source: PLoS Comput Biol. 2023 Nov 29;19(11):e1011658. doi: 10.1371/journal.pcbi.1011658 (PMC10712895; doi:10.1371/journal.pcbi.1011658)
Supplement: S1 Appendix — In-depth description how the experimental envelope projection area used for comparison was calculated from figures depicting biological data published in [1]. (PDF) [file pcbi.1011658.s001.pdf]

S1 Appendix: Calculating envelope projection areas from published data

Sonja Mathias[1*], Igor Adameyko[2,3‡], Andreas Hellander[1‡], Jochen Kursawe[4‡]

[1]Department of Information Technology, Division of Scientific Computing, Uppsala University, Uppsala, Sweden
[2]Department of Physiology and Pharmacology, Karolinska Institutet, Solna, Sweden
[3]Department of Neuroimmunology, Center for Brain Research, Medical University of Vienna, Vienna, Austria
[4]School of Mathematics and Statistics, University of St Andrews, St Andrews, UK

November 16, 2023

* sonja.mathias@it.uu.se

‡ These authors contributed equally to this manuscript as co-senior authors

To estimate biologically realistic values of envelope projection areas we identified clonal envelopes of varying length in Figures of [1]. Specifically, we identified all clonal envelopes across all Figures and supplementary Figures of the paper that were flourescently marked and contained four or five cells, and had a columnar structure. For each such envelope, we manually recorded the centroid location of each contained cell using Fiji's multipoint tool [2] (panel A, Fig AA). The identified cell centre locations were saved in a separate .csv file.

We used these positions to approximate the envelope projection area. First, we fitted a line through all positions. Then, we rotated the coordinate system so that the fitted line was vertical. Since positions in our simulations are measured in cell length, we further rescaled all positions such that the longest vertical distance of the measured clonal envelope of size n was $n-1$, as in a perfectly straight and tightly packed columnar structure there would $n-1$ cell lengths between the cell centres of the furthest-apart cells. Cell centre locations of an example clonal envelope before and after rotating are shown in panel B, Fig AA. Finally, we recorded the width Δx of the clonal envelope in the horizontal direction using the resulting rescaled co-ordinates. This width represents the spread of the clonal envelope in one dimension. In our simulations, envelope projection areas were two-dimensional and calculated by multiplying the

widths of the clonal envelope in the x and the y directions. Hence, we estimated the envelope projection area of clonal envelopes in the experimental images by taking the square of the one-dimensional spread, assuming that the width of a clonal envelope would be similar in both lateral directions.

In practice we implemented this method using a python script that performed the following calculations. First, we subtracted the position of the first recorded cell from all centroid positions so that this cell had co-ordinates (0,0). Then, we fitted a straight line through the centroid positions using the 'polyfit' function of the numpy libraries [3], providing a slope m and y-intercept b. We then transformed all cell centre coordinates so that this line would start at (0,0) and point parallel to the $y-axis$. Specifically, the y-intercept b of the line was subtracted from the y-position of each cell centre and we used the slope m to calculate the rotation angle

$$\Theta = \pi/2 - \arctan(m). \tag{A}$$

Each cell centre position x_i, y_i was then rotated onto the y axis using the rotation transform

$$\begin{pmatrix} \cos(\Theta), -\sin(\Theta) \\ \sin(\Theta), \cos(\Theta) \end{pmatrix} \begin{pmatrix} x_i \\ y_i \end{pmatrix}. \tag{B}$$

Denoting the size of the clonal envelope by n, the position of all coordinates was then multiplied with $(n-1)/y_{\max}$, where $y_{\max}$ is the largest absolute value of all rotated y positions of all cell centres in the dataset. This rescaling achieved that the cell that is furthest away from the origin had the y-coordinate $n-1$, assuring that distances are roughly measured in cell lengths.

Finally, we calculated the width Δx of the clonal envelope by substracting the smallest x-position from the largest x-position in the envelope. We approximated the envelope projection area of the clonal envelope as

$$a = \Delta x^2. \tag{C}$$

This process was applied to a total of 12 clonal envelopes identified in [1] (panel C, Fig AA). The envelope projection areas varied between cells, with a mean of $0.23\,d^2$ and a standard deviation of $0.2\,d^2$.

All images of the form of panel A in Fig AA, all .csv files that have been extracted with Fiji, and the python script used for this analysis are available via our GitHub repository at `https://github.com/somathias/CartilageCBM/`.

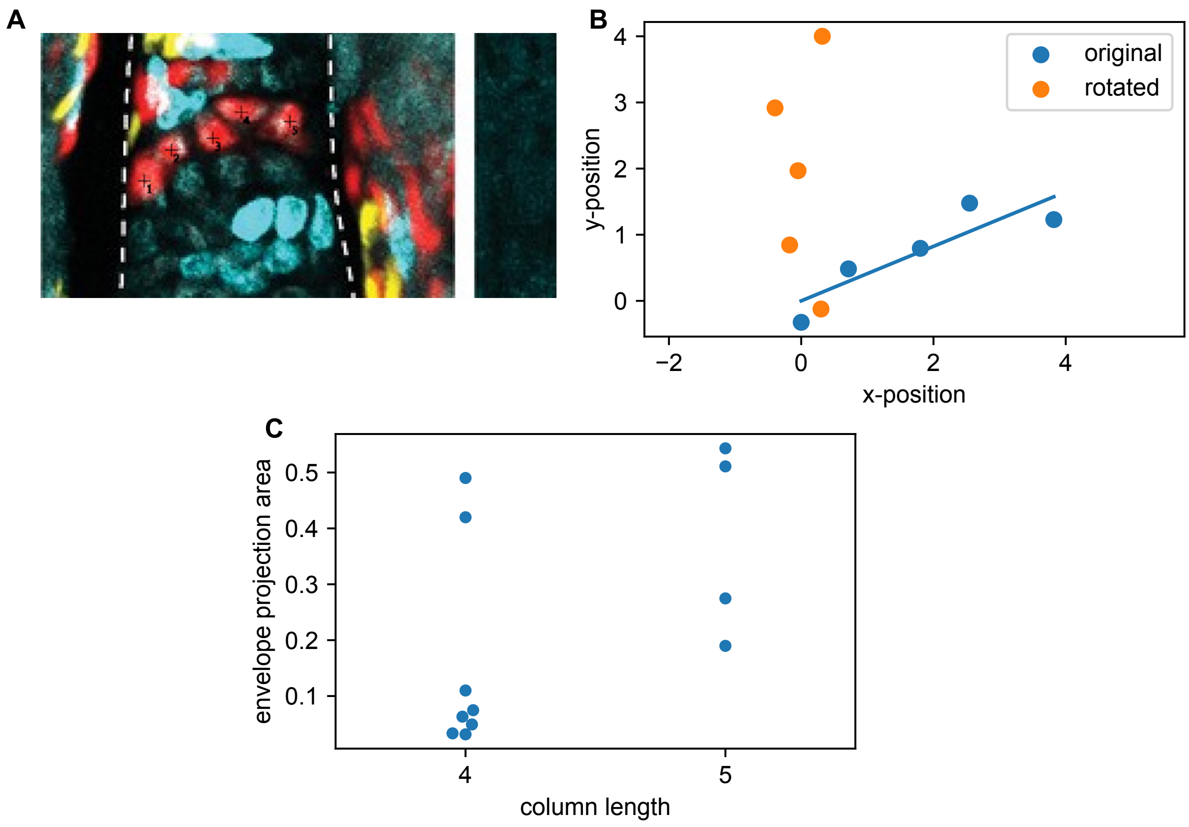

Figure AA: Extracting envelope projection areas from Figures in [1]. A: Cropped section of Fig 4a of [1] with cell centres manually selected in Fiji; B: Blue circles: cell centre positions as extracted from Fiji. Blue line: line fit through the centre positions. Orange circles: rotated cell centre positions. C: All extracted envelope projection areas. Panel A is adapted from Fig 4 in [1], made available under a Creative Commons CC0 public domain dedication (`https://creativecommons.org/publicdomain/zero/1.0/`).

References

[1] Kaucka M, Zikmund T, Tesarova M, Gyllborg D, Hellander A, Jaros J, et al. Oriented clonal cell dynamics enables accurate growth and shaping of vertebrate cartilage. eLife. 2017 Apr;6:e25902.

[2] Schindelin J, Arganda-Carreras I, Frise E, Kaynig V, Longair M, Pietzsch T, et al. Fiji: an open-source platform for biological-image analysis. Nature Methods. 2012 Jul;9(7):676-82. Available from: `https://doi.org/10.1038/nmeth.2019`.

[3] Harris CR, Millman KJ, van der Walt SJ, Gommers R, Virtanen P, Cournapeau D, et al. Array programming with NumPy. Nature. 2020 Sep;585(7825):357-62. Available from: `https://doi.org/10.1038/s41586-020-2649-2`.
