# Supplementary material for: Contributions of cell behavior to geometric order in embryonic cartilage
Source: PLoS Comput Biol. 2023 Nov 29;19(11):e1011658. doi: 10.1371/journal.pcbi.1011658 (PMC10712895; doi:10.1371/journal.pcbi.1011658)
Supplement: Attachment — Submitted filename: Review comments.pdf [file pcbi.1011658.s002.pdf]

Summary of paper

The paper is concerned with a new mathematical model of embryonic cartilage formation, following experimental findings that embryonic cartilage is highly geometrically ordered. A thin sheet is formed by cells which are arranged into columns, and cells in a column share a common ancestor. The sheet grows as a consequence of columns increasing in length, or by insertion of new columns.

The model is a cell-based model which accounts for oriented cell division, mechanical forces between cells as well as some aspects of the surrounding tissue. They investigate several phenomena, with the aim of finding principles that can result in formation of columns of cells, as is observed in vivo.

The authors argue that their work fills a gap that has not been addressed previously, namely, to include both oriented cell division as well as mechanical interactions (repulsive forces) between cells as principles governing embryonic cartilage formation. Both mechanisms have been part of mathematical models previously, but not together.

The model is used to investigate several properties of cartilage formation.

Findings based on simulations:

1. Repulsive forces between cells and unoriented/random cell divisions does not produce column formation.
2. Repulsive forces and oriented cell divisions result in column formations.
3. Increasing the spacing between cells in the initial configuration promotes column straightness.
4. Increased space between cells and the perichondrial boundary promotes column straightness.
5. There is a tradeoff between how long columns are allowed to grow and the straightness of columns. Longer columns are less straight. They argue that this has been observed previously.
6. Sequential increases in the maximum allowed column length promotes order compared to letting columns grow to the same length from the start.
7. Oriented cell division and repulsive forces are not sufficient to form new columns in existing cartilage.
8. New columns can form into existing cartilage provided there is sufficient space to do so.

The model choice is well motivated and have the potential to provide meaningful insights. However, all comparisons to experimental data are qualitative, and no quantitative comparison is made. In my opinion, to demonstrate the utility of the model it should either be directly compared to experimental data or encourage new experiments which can validate the findings of the model. As it stands it can be used to generate and test hypotheses, but it is difficult to determine how credible the findings are. In case such a comparison is not possible, a motivation and discussion of the topic would be suitable.

Can the envelope projection area (or 2D version of it) be obtained from images to get an idea of realistic ranges of this metric? Currently it is difficult to judge which column formations are realistic based only on the envelope projection area of simulations. For instance, I would guess that perfectly aligned columns are as unrealistic as very unordered columns.

Recommended changes to increase readability/quality

1. Suggested changes to the section “Cell proliferation” (starting on line 162)
    - A figure illustrating oriented and random division would be beneficial.
    - The first paragraph is somewhat confusing. It is written that two daughter cells are placed in relation to the mother, as if a division event produced two daughter cells, and the mother cell remains.
    - Consider explicitly writing that the x- and y-coordinates of daughter cells are the same as those of the mother cell (although this can be derived from the description).

2. I am confused about the results in the section “Column growth benefits from distance to the perichondrial boundary”. If I have understood the proliferation mechanism correctly, I cannot see how the given initial conditions can produce anything but perfectly aligned columns when all cells initially have the same z-coordinate, and all daughter cells have the same x- and y-coordinates as their mother cell. Could you explain the cause of misalignment in columns in this case?

3. On lines 113-114 and later on 513-514 you write that you confirm that oriented cell divisions are required/necessary to establish columns. Perhaps this could be reformulated to express that this is one mechanism that indeed result in column formations. As you discuss on lines 556-559, according to experimental data it is not entirely evident that it is oriented divisions that is the/the only cause. On the other hand, it is somewhat implied in a modelling paper that some of the statements relate to the model itself, which is the case here. I would suggest rephrasing the statements to clarify that it is one possible mechanism driving column formation or emphasize that it is a necessary mechanism when using this particular model.

Minor:

1. Missing $$ around a few x,y and z in the text. (e.g. Lines: 216, 239, 240, 340, 561)
2. Double and single quotation marks mixed. (Single marks: Lines: 228, 289, 294)
3. “Centre” and “Center” as used in “Cell-center based…” are mixed throughout the paper.
4. Line 117: Thicker columns sounds like something else, for example having large envelope projection area. Perhaps it could be called longer or taller columns.
5. Lines 168-169: It is written that oriented cell division will be used throughout the paper, even though random orientations are used at one point.
6. Line 185: Repetition of “showed”.
7. Lines 204-205: 72 must be a typo. Shouldn’t there be 8*12=96 cells in the initial configuration?
8. Line 228: Missing “of”: “within the ‘back’ half **of** the tissue..”.
9. Line 357: The word “upwards” is a little bit confusing. I take it this means in the z-direction?
10. Line 359: **A** repulsion-only force

11. Line 401: Consider another formulation to increase clarity of the sentence. Now it could be interpreted as there are two layers in the same simulation.
12. Line 527: Missing "of" in "varying the amount **of** space".
13. Line 542: Typo "compared".
14. Perhaps include a sentence on how the boundary conditions are implemented. If a cell is being pushed across the boundary layer, does the boundary act as a repulsive force of much larger magnitude?
15. Consider moving the description of cells' rest length so that it appears before Eq (3).

Questions

1. Are there other hypotheses in the biological community on how the geometric order occurs? For example, is there any evidence that adhesion or chemotaxis plays a role?

2. Is it possible in imaging studies of cartilage formation to distinguish between cells which have a common ancestor? The reason I am asking is that, in my opinion, it is of importance when choosing a metric of column order. In your choice of envelope projection area, you can keep track of which cells belong to the same envelope. If this is not something that can be done in practice during imaging, another measure that could be applied to both simulations and real images would be more suitable. Something in line with defining a column as a set of cells which all lie within half a cell diameter of each other (in the x-y plane), and not overlapping in the z-direction.
