# Supplementary material for: Contributions of cell behavior to geometric order in embryonic cartilage
Source: PLoS Comput Biol. 2023 Nov 29;19(11):e1011658. doi: 10.1371/journal.pcbi.1011658 (PMC10712895; doi:10.1371/journal.pcbi.1011658)
Supplement: Attachment — Submitted filename: R_Manuscript_PCOMPBIOL-D-23-00028.pdf [file pcbi.1011658.s003.pdf]

The manuscript entitled "Contributions of cell behavior to geometric order in embryonic cartilage" presents a cell-based model for the formation of sheet-like cartilage plates during embryonic development. The model is formulated within the framework of centre-based models, in which cells are represented as spheres that can mechanically interact. The authors aim to investigate how individual cell behaviour of chondrocytes, the cells composing cartilage tissues, results in the geometrical structure of cartilage growing from the condensation of cells, which consists of short monoclonal columns oriented transversally, and how this structure is maintained during cartilage lateral expansion and thickening.

The manuscript is well-written and easy to follow, with a clear structure and informative figures. The authors also provide code for all simulations, which is a valuable resource. However, I have concerns about the rigidity of the model, which lacks a connection to actual dimensions and experimental data. The simulation setups are relatively inflexible and tend to produce expected results, such as the monoclonal columnar structure of the cartilage sheet resulting from the initial honeycomb pattern and strictly transverse cell division between two rigid boundaries. As a result, it is not clear what new insights this work provides into cartilage formation.

To improve the manuscript, I recommend that the authors strengthen the connection between their modelling results and experimental observations. If experimental data is unavailable, they should consider performing parameter sweeps or sensitivity analyses to identify the key parameters driving their model's behaviour. Additionally, the authors should address the issues listed below.

Major issues:

1. Could the authors clarify several aspects of their choice of the cell division model? In particular:
   - G1 is typically the most variable in terms of duration and longest cell cycle phase. Could the authors clarify why the G1 duration was assumed to be fixed to 3 a.u., whereas the G2 duration is an exponentially distributed random variable with a longer mean value of 10 a.u.? What about the S and M phases?
   - What is the parameter of the exponential distribution assumed for the G2 duration? In Table 1, I believe that 10 a.u. is the mean value. Could this be clarified within the manuscript?
   - Could the authors clarify their choice of the cell cycle model (i.e. the exponential distribution)?
   - The work by Kaucka et al. ([1] within the manuscript) provides data on the distribution of division orientation angles. Can these data be used for model calibration? Another question that could be investigated using the framework of the model is how spread the distribution of division orientation angles (its variance) can be around 90° so that the geometrical columnar structure is still observed (also can columns be 'straightened' by stronger repulsion force?).
   - Does the strength of activation by BMP influence how much cell divisions are aligned with the transversal direction? Can this be investigated using this model?

2. The current model assumes that the influence of the ECM (extracellular matrix) on chondrocyte behaviour can be implicitly captured by setting an appropriate friction coefficient. However, this approach may not be sufficient in three-dimensional environments where cells are embedded in a fibrous ECM mesh with mechanical properties. In such scenarios, it is unclear how chondrocytes invade the ECM and whether they can degrade the surrounding matrix through proteolysis.

   The authors suggest that when ECM is present in the gaps between cells, monoclonal columns are more transversally oriented. However, this conclusion seems questionable given the pre-imposed honeycomb pattern and the increased spacing between cell centres at $t = 0$, which decrease cell-to-cell mechanical interactions and would be expected to result in vertically oriented columns. Furthermore, the numerical experiment of column insertion in place of a deleted column yields predictable results that provide little insight. Additionally, if such large spaces filled with ECM were available for column insertions, they would likely be visible in experimental results (e.g. Figures from Kaucka et al. [1]).

Minor issues:

3. On line 119, "not sufficient" is perhaps too strong since no sensitivity analysis or model calibration was presented. The authors might want to soften this sentence.

4. Can the authors clarify somewhere within the manuscript how the second (boundary) perichondrial cell appears? Or both perichondrial cells are present at cartilage sheet boundaries, and both divide?

5. Can the authors explain their choice of the initial honeycomb arrangement of cells in the x-y plane? This setup makes sense for the first numerical scenario where the authors test whether random division orientation combined with regular cell positioning is sufficient to generate monoclonal columns. However, for later numerical experiments, this might be an unnecessary restriction (even if slightly perturbed). Are there some experimental data available for mesenchymal condensations?

6. On line 407 and also in Figure 6 and its caption, the coordinate of the midsection should be $z = \frac{u+l}{2}$ instead of $z = \frac{u-l}{2}$ (although, I understand that since $l = 0$, this is not going to change simulation results).

7. I would like to suggest using the same text in (the beginning of) captions of Figures 2 and 3 (illustrating the two initial conditions used in simulations) as in the titles of the subsections, which introduce the corresponding initial condition (and refer to these initial conditions in a similar way, e.g. Configuration (i)).

8. Around line 420, could the authors clarify whether for the numerical experiments of tissue thickening u is adjusted at the time, $t = 0$, or it is gradually increased during simulations?

9. Can stronger repulsion force contribute to column straightening (in general and in experiments of tissue thickening and lateral expansion by column insertion)?

10. Could the authors clarify the sentence on lines 227-228?

11. On line 473, it is indicated that 10 cells were activated to divide for the column insertion experiment. However, I see only 9 activated cells in Figure 9.

12. On line 244, the authors state that perichondrial cells divide asymmetrically, but on lines 475-476, the division of perichondrial cells in the numerical experiment with column insertions is symmetrical. Is the division of perichondrial cells context-dependent? Could the authors clarify this within the text?

13. Could the authors add a reference supporting their statement that "cell adhesion molecules are not present" in chondrocytes (lines 158-159)? (Is it the same work cited in the following sentence?)

14. In the code of the model shared by the authors on GitHub, I would like to suggest that the project name `cartilage` in all `CMakeLists.txt` files (`chaste_do_apps_projects(cartilage)`) is changed to `CartilageCBM` as the name of the GitHub repository (otherwise, users will have to do it themselves to be able to compile and run the code).
