# Supplementary material for: Contributions of cell behavior to geometric order in embryonic cartilage
Source: PLoS Comput Biol. 2023 Nov 29;19(11):e1011658. doi: 10.1371/journal.pcbi.1011658 (PMC10712895; doi:10.1371/journal.pcbi.1011658)
Supplement: Attachment — Submitted filename: response_to_reviewers_comments20230926.pdf [file pcbi.1011658.s004.pdf]

Submission of the revised version of our manuscript (PCOMPBIOL-D-23-00028) and our responses to the reviewers' comments

Dear Professor Maini and Professor Alber,

With this letter I am submitting the revised version of our manuscript "Contributions of cell behavior to geometric order in embryonic cartilage" (manuscript number PCOMPBIOL-D-23-00028) to PLOS Computational Biology.

We are grateful for the thorough review and the insightful suggestions to improve the paper. We have carefully revised the manuscript, addressing all comments raised by reviewers. In particular, our new manuscript now includes an estimation of our order parameter, the envelope projection area, from experimentally measured images. In addition, we added a new results section 'Column order is sensitive to perturbations in the division orientation', in which we analyze the effect of small deviations in division orientation onto the order parameter.

Below you find our detailed responses to the reviewer comments, which are reproduced here in italic font. Our responses follow in blue upright font. Page and line numbers refer to the attached revised manuscript in which all changes to the text are highlighted in bold font.

All authors have approved the revised manuscript and agree with its submission to PLOS Computational Biology.

As corresponding author,
Sonja Mathias

Reviewer #1:

Summary of paper

The paper is concerned with a new mathematical model of embryonic cartilage formation, following experimental findings that embryonic cartilage is highly geometrically ordered. A thin sheet is formed by cells which are arranged into columns, and cells in a column share a common ancestor. The sheet grows as a consequence of columns increasing in length, or by insertion of new columns.

The model is a cell-based model which accounts for oriented cell division, mechanical forces between cells as well as some aspects of the surrounding tissue. They investigate several phenomena, with the aim of finding principles that can result in formation of columns of cells, as is observed in vivo.

The authors argue that their work fills a gap that has not been addressed previously, namely, to include both oriented cell division as well as mechanical interactions (repulsive forces) between cells as principles governing embryonic cartilage formation. Both mechanisms have been part of mathematical models previously, but not together.

The model is used to investigate several properties of cartilage formation.

Findings based on simulations:

1. *Repulsive forces between cells and unoriented/random cell divisions does not produce column formation.*
2. *Repulsive forces and oriented cell divisions result in column formations.*
3. *Increasing the spacing between cells in the initial configuration promotes column straightness.*
4. *Increased space between cells and the perichondrial boundary promotes column straightness.*
5. *There is a tradeoff between how long columns are allowed to grow and the straightness of columns. Longer columns are less straight. They argue that this has been observed previously.*
6. *Sequential increases in the maximum allowed column length promotes order compared to letting columns grow to the same length from the start.*
7. *Oriented cell division and repulsive forces are not sufficient to form new columns in existing cartilage.*
8. *New columns can form into existing cartilage provided there is sufficient space to do so.*

The model choice is well motivated and have the potential to provide meaningful insights. However, all comparisons to experimental data are qualitative, and no quantitative comparison is made. In my opinion, to demonstrate the utility of the model it should either be directly compared to experimental data or encourage new experiments which can validate the findings of the model. As it stands it can be used to generate and test hypotheses, but it is difficult to determine how credible the findings are. In case such a comparison is not possible, a motivation and discussion of the topic would be suitable.

We thank the reviewer for their insightful comments.

We agree with the reviewer that computational models gain value through comparison with existing experiments, and by informing new experiments. Our manuscript includes such comparisons and predictions throughout the result section:

(i) The first result of the paper reported that oriented cell divisions are required to enable column formation. This agrees well with experimental findings that cell divisions of chondrocytes are indeed oriented, and that perturbing the division orientation destroys columnar order. This connection was not clear in the original manuscript, and we have now included a new paragraph on page 17, lines 379ff to highlight this.

(ii) Our model predicts that longer columns are more disordered than shorter columns. For this prediction we do not have quantitative data available, but visual inspection of published figures from Kaucka et al (2017) indicates that the prediction is correct. We point out the relevant figure panels of this paper on page 24 line 539 of the revised manuscript. In these figure panels, it is visible that cell ordering in thicker tissue cross sections appears more disordered than those in thinner cross sections. Unfortunately, it is not possible to quantify these differences from the published data from Kaucka et al., as they do not contain fluorescently marked clonal envelopes of sufficient size.

(iii) Our investigation on iterative growth (Figure 10 of the revised manuscript) highlights that iterative growth is beneficial for column order. In Kaucka et al, such iterative growth was indeed reported. Our model hence suggests that these biological dynamics may be functionally important for column order, which we would not have known without the computational experiment.

(iv) Our findings on column insertion can be used to motivate further experiments. In particular, the model highlights that our key modeling assumptions on cell-cell interactions (forces are repulsive and cell divisions are oriented) are insufficient to explain tissue growth, and other factors are required. This insight would not have been available without our computational investigation and can be used to guide further experimentation. In particular, the model can be used to test feasible hypotheses *in silico.* For example, our simulations in Figure 11 of the revised manuscript suggest that deposition of extracellular matrix may be a component of column insertion. This can be tested by performing experiments that fluorescently label extracellular matrix, or measure matrix secretion. We have added a sentence to the revised manuscript on page 28, line 617 to highlight this.

(v) In response to Reviewer 3 below we added a new results section to the revised manuscript, ‘Column order is sensitive to perturbations in division orientation’, in which we add random noise to simulated division orientations and relate the findings to measurements on cell alignment distributions of clonal cells from Kaucka et al. (page 18, lines 403ff of the revised manuscript).

Can the envelope projection area (or 2D version of it) be obtained from images to get an idea of realistic ranges of this metric? Currently it is difficult to judge which column formations are realistic based only on the envelope projection area of simulations. For instance, I would guess that perfectly aligned columns are as unrealistic as very unordered columns.

Getting such data is a great suggestion, and we have included this analysis in the revised manuscript on page 17, lines 388 ff. These data quantitatively confirm that the reviewer is

correct in stating that perfectly aligned columns are unrealistic. The experimentally measured envelope projection area in clonal envelopes containing 4 or 5 cells is estimated to be 0.23±0.2. This is indeed larger than simulated envelope projection areas for highly ordered columns (value 0.04±0.02 at t=80 a.u. in Figure 5d), while remaining noticeably smaller than the simulated value for disordered columns in our simulations (value 1.31±0.06 at t=80a.u. in Figure 5d).

Recommended changes to increase readability/quality

1. *Suggested changes to the section "Cell proliferation" (starting on line 162)*
    a. *A figure illustrating oriented and random division would be beneficial.*
    We have included such a figure as the new Figure 2 on page 7 of the revised manuscript, which is described in the subsection 'Cell proliferation' on page 7, lines 166.
    b. *The first paragraph is somewhat confusing. It is written that two daughter cells are placed in relation to the mother, as if a division event produced two daughter cells, and the mother cell remains.*
    Thank you for spotting this. We have edited the text on page 7 of the revised manuscript and included a sentence clarifying that the mother cell is removed from the simulation.
    c. *Consider explicitly writing that the x- and y-coordinates of daughter cells are the same as those of the mother cell (although this can be derived from the description).*
    We decided to include this and have added a sentence to the revised 'Cell proliferation' section.
2. *I am confused about the results in the section "Column growth benefits from distance to the perichondrial boundary". If I have understood the proliferation mechanism correctly, I cannot see how the given initial conditions can produce anything but perfectly aligned columns when all cells initially have the same z-coordinate, and all daughter cells have the same x- and y-coordinates as their mother cell. Could you explain the cause of misalignment in columns in this case?*

We thank the reviewer for pointing out that the manuscript was unclear about this. Cells in our initial configuration are not located on a perfect hexagonal lattice in the x-y direction. Instead, we add small random perturbations to each cell center as part of generating initial conditions for our simulations. This avoids the type of artifacts that one would expect from a perfectly ordered initial condition and which the reviewer highlights. Since cells are not perfectly ordered, cells in emerging columns from separate clonal envelopes end up pushing against each other in our simulations, which can lead to disorder.

We addressed this comment in the revised manuscript by adding clarifying sentences to the described paragraph on page 21, line 461, and to the caption of Figure 7.

On lines 113-114 and later on 513-514 you write that you confirm that oriented cell divisions are required/necessary to establish columns. Perhaps this could be reformulated to express that this is one mechanism that indeed result in column formations. As you discuss on lines 556-559,

according to experimental data it is not entirely evident that it is oriented divisions that is the/the only cause. On the other hand, it is somewhat implied in a modelling paper that some of the statements relate to the model itself, which is the case here. I would suggest rephrasing the statements to clarify that it is one possible mechanism driving column formation or emphasize that it is a necessary mechanism when using this particular model.

This is a good point. We have addressed this comment by changing the sentence to 'Similar to previous experimental findings [1], our model requires oriented cell divisions to establish columnar order in embryonic cartilage.' on page 5, line 114 of the revised manuscript.

Minor:

Thank you for spotting all of these!

1. *Missing $$ around a few x,y and z in the text. (e.g. Lines: 216, 239, 240, 340, 561)*

We fixed these in the revised manuscript.

2. *Double and single quotation marks mixed. (Single marks: Lines: 228, 289, 294)*

We fixed these in the revised manuscript.

3. *"Centre" and "Center" as used in "Cell-center based..." are mixed throughout the paper.*

We changed these to 'centre' throughout.

4. *Line 117: Thicker columns sounds like something else, for example having large envelope projection area. Perhaps it could be called longer or taller columns.*

We have changed this to 'longer' columns.

5. *Lines 168-169: It is written that oriented cell division will be used throughout the paper, even though random orientations are used at one point.*

This sentence now reads 'Throughout the paper, we will use vertically oriented cell divisions as in Figure 2b, except for two cases: the first computational experiment in the Results section where we specifically study the effect of a random division orientation as in Figure 2a, and the second computational experiment where we study the effect of perturbations added to vertical division orientations (details explained in place).'

6. *Line 185: Repetition of "showed".*

We now fixed this.

7. *Lines 204-205: 72 must be a typo. Shouldn't there be 8*12=96 cells in the initial configuration?*

This is correct, there are indeed 96 cells in the initial configuration. We have now fixed this typo in the manuscript.

8. *Line 228: Missing "of": "within the 'back' half of the tissue..".*

We have now fixed this.

9. *Line 357: The word "upwards" is a little bit confusing. I take it this means in the z-direction?*

Yes, we have clarified this in the manuscript.

10. *Line 359: A repulsion-only force*

We fixed this in the revised manuscript.

11. *Line 401: Consider another formulation to increase clarity of the sentence. Now it could be interpreted as there are two layers in the same simulation.*

We clarified this by removing ‘at two different heights’ from this sentence.

12. *Line 527: Missing “of” in “varying the amount of space”.*

We fixed this in the revised manuscript.

13. *Line 542: Typo “compared”.*

We fixed this in the revised manuscript.

14. *Perhaps include a sentence on how the boundary conditions are implemented. If a cell is being pushed across the boundary layer, does the boundary act as a repulsive force of much larger magnitude?*

We addressed this by adding the following sentence underneath equation 4 on page 15 of the revised manuscript: ‘If the new position x_i^{new} is beyond one of the rigid boundaries of the domain, it is projected back onto the boundary by changing its z-component to the z-position of the boundary.’

15. *Consider moving the description of cells’ rest length so that it appears before Eq (3).*

Thank you. However, we hope it will be OK to not follow this small suggestion. We do not believe this would help the readability of the text. If we were to follow the suggestion, one parameter in equation 3 would be defined before the equation, while the other parameters would be named after the equation. This would be inconsistent.

Questions

1. *Are there other hypotheses in the biological community on how the geometric order occurs? For example, is there any evidence that adhesion or chemotaxis plays a role?*

This is not a well-researched topic. Kaucka et al (2017) were the first authors to report columnar structures in cartilage formation, and since then no other authors have investigated the mechanisms further. However, the data in Kaucka et al (2017) as well as previous literature allow for some speculation.

We do not believe cell-cell adhesion to play a role in column formation. In figures from Kaucka et al (2017), an example of which is shown in Figure 1B of the revised manuscript, there are visible gaps between cell outlines, demonstrating that there is extracellular space between cells. Additionally, Oberlender et al. (1994) showed in chick-embryos that N-Cadherin, which is an adhesion molecule used in cell-cell contact, is expressed in mesenchymal condensations, but not in mature chondrocytes, thus further suggesting that chondrocytes do not adhere to each other.

Regarding chemotaxis, it has been shown that chemotaxis is inducible in human adult chondrocytes, and this is believed to play a role in bone injury repair (Mishima et al., 2008). Mishima et al (2008) showed that this induction can indeed happen in response to BMP proteins, which we know provide a transversal gradient in cartilage plates (Kaucka et al, 2017). Hence, there is a possibility that reported oriented cell divisions in Kaucka et al. (2017) may not represent direct orientation of the mitotic spindle, but instead cell divisions may be oriented randomly, and daughter cells subsequently use the BMP gradient to arrange themselves along the z-axis using chemotaxis. This would be supported by the fact that chondrocytes are known to express integrins (Dieterle et al, 2021), which are molecules responsible for cell-matrix

adhesion, showing that chondrocytes may adhere to extracellular matrix. However, we do not know of studies reporting chemotaxis of chondrocytes during development in plate-like structures in which columnar clonal envelopes are present.

Overall, these are interesting points, and we have added them to the discussion of our revised manuscript (page 30, lines 648ff, page 31, lines 678ff and page 32, lines 703ff).

2. *Is it possible in imaging studies of cartilage formation to distinguish between cells which have a common ancestor? The reason I am asking is that, in my opinion, it is of importance when choosing a metric of column order. In your choice of envelope projection area, you can keep track of which cells belong to the same envelope. If this is not something that can be done in practice during imaging, another measure that could be applied to both simulations and real images would be more suitable. Something in line with defining a column as a set of cells which all lie within half a cell diameter of each other (in the x-y plane), and not overlapping in the z-direction.*

Yes, this is possible. Data reported in Kaucka et al (2017) specifically included information on clonal envelopes, i.e. sets of cells that have a common ancestor. For example, in Figure 1B of the revised manuscript, which is from Kaucka et al (2017), clonal envelopes have been fluorescently labeled in yellow, red, and blue. The fact that this previous study includes these data motivated our use of the envelope projection area as a measure for column order. We hope that this is clearer in the revised manuscript, which includes the above-mentioned new paragraph in the results section in which envelope-projection areas are quantified from Kaucka et al. (2017) (see page 17, lines 388ff).

Reviewer #2:

Re: Review "Contributions of cell behaviour to geometric order in embryonic cartilage"
--

This article develops a three-dimensional model investigating the formation of columnar structure in early cartilage (and bone) formation during organism development. The model is motivated from a separate experimental / modelling paper (the model there was a cellular automaton on a lattice). In detail, the authors consider two scenarios: (1) the emergence of columns from dense collections of mesenchymal cells; and (2) the insertions of additional columns into an existing columnar structure (e.g. tissue growth).

The authors goal is to elucidate the effect mechanical forces and cell division play in this process. For this purpose they propose a simple centre based model implemented in Chaste, with isotropic cell-medium friction, and cell-cell repulsion as the only cell-cell forces. Cells are placed in a three dimensional rectangle with a solid repulsive boundary placed below and

above. The model setup resembles as cross-section of the modelled tissue configuration. Since previous modelling for this system was done using a lattice based model, mechanical insights were limited, which is being addressed in this article.

Through simulation studies the authors demonstrate that cell-cell repulsion with oriented cell division along the z-direction is sufficient for columnar structure to form, from an initially condensed group of cells (fairly flat). Further the authors demonstrate that oriented cell division is required, without columns do not form. The "columnarness" of their simulation outputs is quantified using an adequate order parameter. In addition, the authors investigate the effect and role other key parameters in their model play. Finally, the authors investigate whether their model supports the insertion of additional column. I suspect the motivation is to investigate what happens in a growing tissue? Their model cannot recapitulate the insertion of novel columns, hinting that additional mechanisms are required.

The paper is well written, all details are included, the code is available, and I feel confident that the results are reproducible. In my opinion this article provides a simple mechanical explanation for the formation of the observed columnar structure, the article clearly demonstrates limitations of their model, which highlight areas of future investigation. I do have some small remarks, and questions below, but I expect those to be easily addressable, and following revisions recommend this article for publication.

We thank the reviewer for their supportive comments.

Comments
----

1. *I have three questions regarding the model for cell-cell division. As far as I understand cell division is implemented as follows. When the cell cycle time is completed two cells are placed a given distance apart along a given orientation.*
    a. *Q1: Is the volume of the cells increased as they progress through the cell cycle?*

No, the volume of the cells is not increased during the cell cycle. We added a clarifying sentence about this on page 9 the revised manuscript (line 194).

    b. *Q2: Does simply placing the cells a distance r_0 apart immediately not lead to simulation challenges? If I were to implement this, I would let the cell double volume, then place two cells of the same volume at the same location, and then slowly push them apart to avoid numerical integration problems, and keep the system close to mechanical equilibrium. Is this not required since the cell population is always somewhat sparse?*

We agree with the reviewer that placing new daughter cells far apart from each other would lead to numerical inaccuracies. We argue that that the effect of such inaccuracies is minimal in our simulations, since we choose a small value for r_0 (r_0=0.3), i.e. newly formed daughter cells are placed close to each other and then push away from one other until a mechanical equilibrium is achieved. While this may still lead to high forces immediately after cell division, we

believe these forces will not strongly affect the equilibrium position that is eventually found. This algorithm for cell division implementations is common in multiple widely-used software frameworks for cell-centre based models, such as Chaste (Cooper et al, 2020) and PhysiCell (Ghaffarizadeh et al, 2018). A more in-depth discussion of the literature on this topic is available in Section 2.5 of Mathias et al (2020), reference [35] of the revised manuscript.

We added a short paragraph on page 30, lines 661ff to address this question.

c. *Q3: In the simulations in which columns form, the division orientation is always along the z-axis i.e. fixed. Have the authors considered mechanical re-orientation of the cell division axis? In other words, let the mechanical interactions of the dividing cell determine the cell division axis? Or are the authors convinced that only the gradient of BMP guides the division axis?*

We have not considered such a mechanical re-orientation of the division axis. We are indeed convinced that only the BMP gradient guides the division axis, since Kaucka et al (2017) provided experimental evidence that this is the case. Specifically, they performed experiments in which a cell-surface receptor for BMP, ACVR1, was overactivated in individual cells. This affected the ability of cells to interpret the local BMP gradient and led to mis-oriented cell divisions. If the division orientation was mechanically guided, perturbing the receptor for BMP would not have been sufficient to perturb the division direction. We apologize that this connection to experimental evidence was not clear in the original manuscript, and have included a paragraph on it in the revised manuscript (page 17, lines 379 ff).

2. *This comment is more of an observation. The authors demonstrate that oriented cell division is not sufficient to explain the intercalation of new columns into a pre-existing structure, unless sufficient space is available. This somewhat reminds me of classical Turing system results on growing domains see for instance Crampin et. al. (1999) i.e.\ a new spike or stripe can be formed when sufficient space is available in the domain to accommodate it. Similarly, with the coarsening phenomena linked to growth (although there I do not completely understand the difference between sequential and continuous growth).*

We thank the reviewer for this insightful comment. Unfortunately, we do not see an obvious way to incorporate this into our manuscript. While there is a clear analogy here in which available space accommodates a larger pattern, the dynamics of a Turing system are inherently different from our reported findings. Specifically, interactions between peaks in a Turing pattern are biochemical, whereas our cell-cell interactions are mechanical.

3. *Line 150: I follow the authors argumentation regarding why only a repulsive cell-cell interaction potential is used. But why is this interaction potential quadratic. Why not for instance, use a Hertz interaction potential or yet a different power? The authors finally explain their choice in the discussion. In my opinion it would make sense to explain their decision in the method or at least refer the reader to the discussion.*

To address this comment we have added the following sentences to the Methods section on page 7, line 163: ‘We do not expect the exact shape of the force function (3) to affect our

results. This is based on previous findings in [35] and will be discussed further in the Discussion section.'

4. *I'm confused about with the authors description of the proliferation control. In detail, the paragraph beginning on line 184. Does this mean each cell can only divide a maximum of four times? If not how is the clonal envelope defined and computed?*

The limit is imposed on the size of a clonal envelope. I.e. when a clonal envelope reaches the size n_max, then cells in the clonal envelope cease to divide. This is different from allowing four cell divisions, as four rounds of cell division would lead to 2^4 = 16 cells in total, which would be biologically unrealistic.

We clarified the definition of clonal envelopes in the manuscript by moving the paragraph that the reviewer mentions into its own subsection 'Definition of clonal envelopes'. It now starts with the sentences: 'We call the collection of all cell offspring arising from one ancestor cell that is present in the initial configuration a *clonal envelope*. That ancestor cell may be a mesenchymal or perichondrial cell, see section 'Initial and boundary conditions' below.'

5. *Clonal envelopes are referred to many times before they are defined. It's confusing.*

Clonal envelopes are now defined in the new subsection mentioned in the previous response. The term 'clonal envelope' is not mentioned before this subheading in the revised manuscript.

6. *On line 218 the authors state that they do not impose any boundary conditions in the xy-plane. Does this mean that periodic boundary conditions are imposed?*

No, this means cells are allowed to move into any position in the x-y plane. Since our model does not involve PDEs and the dynamics are governed by force laws acting between a finite set of points, further boundary conditions are not necessary. In practice, spreading of the cell population in the x-y direction is limited due to an absence of forces promoting such spreading, and due to the presence of friction forces which naturally oppose cell movement. We have added a clarification of this after the sentence the reviewer mentions, on page 11, line 233 of the revised manuscript.

7. *Lines in Figure 4d, 5d, 6d, 7d are not distinguishable in BW or for people with color seeing challenges. I would encourage the authors to employ different linestyles to avoid that challenge.*

We thank the reviewer for pointing this out. All figures now employ different line styles where applicable to help distinguish between lines belonging to different parameter settings.

8. *Line 380: The authors hypothesize that column formation is aided by increased distance due to reduced mechanical repulsive forces. Would it be possible to quantify this interpretation?*

Yes, that is possible. For example, to quantify the difference in forces that we would expect between the scenarios where c=1.0 and c=1.1 in Figure 6d of the revised manuscript, we could consider the maximal force two cells may experience in the initial configuration for each scenario. Since our repulsive force increases with smaller distances between cells, this requires evaluating the closest distances two cells can have in these initial configurations. Taking our

implemented perturbations to a perfect hexagonal lattice into account, the closest distance that two cells can have to each other in the initial configuration is 0.8 when c = 1.0, and it is 0.9 when c = 1.1. Applying these distances into the force law (equation 3) reveals that when c=1.1, the maximal force that two cells experience between each other in the initial configuration is four times smaller than the maximal force that the two cells may experience when c = 1.0.

We have added this interpretation to the same paragraph of the revised manuscript, on page 21 lines 475 ff.

9. *Line 382: Which is the perichondrial boundary again? Maybe just reiterate this as many readers will have forgotten at this point i.e.\ that this is a discussion on the mechanical forces generated by planes located at z = u, l.*

To address this comment, we have added a clarifying sentence below the heading that the reviewer mentions, on page 20, line 436 of the revised manuscript.

Reviewer #3:

The manuscript entitled "Contributions of cell behavior to geometric order in embryonic cartilage" presents a cell-based model for the formation of sheet-like cartilage plates during embryonic development. The model is formulated within the framework of centre-based models, in which cells are represented as spheres that can mechanically interact. The authors aim to investigate how individual cell behaviour of chondrocytes, the cells composing cartilage tissues, results in the geometrical structure of cartilage growing from the condensation of cells, which consists of short monoclonal columns oriented transversally, and how this structure is maintained during cartilage lateral expansion and thickening.

The manuscript is well-written and easy to follow, with a clear structure and informative figures. The authors also provide code for all simulations, which is a valuable resource. However, I have concerns about the rigidity of the model, which lacks a connection to actual dimensions and experimental data.

We apologise that the connection to data and biological detail was not sufficiently clear in the original manuscript. A full response to this concern is available in our reply to Reviewer 1 (top comment), who made a similar point.

In brief, we have related our model to biological observations throughout the results section. For example, the fact that oriented cell divisions are required to enable column formation is confirmed by biological experiments. Similarly, the prediction that longer columns are more disordered can be observed in biological data. Additionally, we strengthened the connections between the model and the data in the revised manuscript. For example, we added a new paragraph and supplementary material for estimating envelope projection areas from biological

imaging data. Further examples and clarifications are listed in the above-mentioned response to Reviewer 1.

The simulation setups are relatively inflexible and tend to produce expected results, such as the monoclonal columnar structure of the cartilage sheet resulting from the initial honeycomb pattern and strictly transverse cell division between two rigid boundaries. As a result, it is not clear what new insights this work provides into cartilage formation.

It is not clear to us in which sense our simulation setups are inflexible, as our manuscript reports results from eight different computational experiments, including multiple parameter variations.

The result that 'the monoclonal columnar structure of the cartilage sheet resulting from the initial honeycomb pattern and strictly transverse cell division between two rigid boundaries' is only one of multiple results sections. Our paper further analyses the influence of space in the initial condition, the effect of clone size, the effect of biologically observed iterative growth, and mechanisms for column insertion.

While some of our results may feel 'expected' to the reader, we argue that we would not know the outcome of our simulations without running them. Additionally, even if we ought to expect our simulation results, we would not have formed that expectation without the process of modelling itself. The predictions from our model are biologically insightful and can be used to guide future biological investigation. This is why we seek to publish them.

For the convenience of the reviewer and editor, we reiterate the new and biologically insightful key results of our paper here:

(i) While the requirement for oriented cell divisions to form columnar clonal envelopes has been known, it is insightful to know that this requirement is also predicted in an off-lattice simulation considering mechanical interactions.

(ii) Our simulations predict that spacing between progenitor cells in the initial mesenchymal condensation will influence column straightness.

(iii) Similarly, the initially available space between the perichondrial boundaries is predicted to play a role in column order.

(iv) Our model correctly predicts that larger clonal envelopes are more disordered.

(v) Iterative growth of columns improves column order. This iterative growth has been reported by Kaucka et al (2017) and it was suggested that it's functional role is to control the dimension of the tissue as a whole, e.g. the thickness of a cartilage sheet can be uniformly increased by adding one cell to each clonal envelope. Our simulations suggest that such iterative growth also maintains the order of columns, which is a new insight.

(vi) In response to a further comment from Reviewer 3 below, we measured how perturbations in the division orientation can affect column order, and related these results to measurements of angle distributions in Kaucka et al.

Results (i-vi) illustrate that very simple assumptions (oriented cell division and purely repulsive cell-cell interactions) are sufficient to explain the formation of columnar structures in cartilage sheets from mesenchymal condensations.

(vii) These simple assumptions are insufficient to explain lateral growth of the tissue through column insertion, and further biological mechanisms will be required to enable such column insertion. This is another new biological insight. Our simulation suggests that the deposition of extracellular matrix before column insertion may be a missing component.

To improve the manuscript, I recommend that the authors strengthen the connection between their modelling results and experimental observations. If experimental data is unavailable, they should consider performing parameter sweeps or sensitivity analyses to identify the key parameters driving their model's behaviour. Additionally, the authors should address the issues listed below.

We have strengthened the connection between our modelling results and experimental observations in the revised manuscript and outlined the changes in our response to Reviewer 1's first comment above.

We agree that parameter sweeps and sensitivity analyses are important to identify key parameters driving model behaviour. This is why parameter variations were a core component of the original manuscript (initial lateral spacing in Figure 7, initial vertical spacing in figure 8, total envelope size in Figure 9 in the revised manuscript). We have extended the revised manuscript with a further analysis on division angle orientation (Figure 6), which also contains a parameter variation (see full description below).

Major issues:

1. *Could the authors clarify several aspects of their choice of the cell division model? In particular:*
    a. *G1 is typically the most variable in terms of duration and longest cell cycle phase. Could the authors clarify why the G1 duration was assumed to be fixed to 3 a.u., whereas the G2 duration is an exponentially distributed random variable with a longer mean value of 10 a.u.? What about the S and M phases?*

We apologise that this was unclear in the original manuscript. It was misleading to refer to the two cell-cycle phases in our model as G1 and G2 phase. It was not our intention to represent each biological phase of the cell cycle. Our cell cycle model simply makes the assumption that the cell cycle will be longer than a minimal duration, but is otherwise randomly distributed.

We addressed this comment by changing Table 1 on page 13 of the revised manuscript to not refer to the cell-cycle phases as G1 and G2. Additionally, we included the values of the two

durations in the paragraph in which they are described (line 189ff on page 8 of the revised manuscript). This paragraph now reads

‘To determine the timing of cell division events, we employ a cell cycle model containing two phases. The first cell cycle phase has a fixed duration g1 = 3.0a.u., while the duration of the second cell cycle phase is exponentially distributed with mean g2 = 10a.u. This choice of cell cycle model prevents synchronicity of cell divisions and thus ensures that neighboring cells do not divide simultaneously. This helps to maintain the quasistatic limit of our simulations (see discussion below). Similarly, including a fixed duration phase prevents immediate successive divisions. The volume of a cell does not change during the cell cycle.’

b. *What is the parameter of the exponential distribution assumed for the G2 duration? In Table 1, I believe that 10 a.u. is the mean value. Could this be clarified within the manuscript?*

This is addressed in the above-mentioned paragraph.

c. *Could the authors clarify their choice of the cell cycle model (i.e. the exponential distribution)?*

This is addressed in the above-mentioned paragraph.

d. *The work by Kaucka et al. ([1] within the manuscript) provides data on the distribution of division orientation angles. Can these data be used for model calibration?*

This is a great suggestion. The data by Kaucka et al indicate that division orientations may vary stochastically, with a standard deviation of up to 15° from the vertical direction. Based on these measurements, we designed new computational experiments in which we added small perturbations to the vertical division orientation (new section ‘Column order is sensitive to perturbations in division orientation’, lines 403ff of the revised manuscript). In brief, we found that even for small random perturbations in division orientation (<1°), column order was affected. Intriguingly, the envelope projection area our simulations predict for such small perturbations of around 1° or 3° is closer to experimentally observed values than when perturbations are neglected (see manuscript for details). As the size of perturbations increases, column order decreases.

To fully answer the question, we believe the angle distributions measured by Kaucka et al. cannot be used directly to calibrate the size of the variation in the division angle. These division angles are obtained using ‘snapshot’ imaging experiments after EdU incorporation, i.e. they are recorded at unknown time intervals after a division occurred. Thus, they measure the compound effects of mitotic spindle orientation and daughter cell displacements after division. Nonetheless, the measurements provide an upper ‘ballpark’ estimate of what amount of perturbations may be realistic.

e. *Another question that could be investigated using the framework of the model is how spread the distribution of division orientation angles (its variance) can be around 90◦ so that the geometrical columnar structure is still observed (also can columns be ‘straightened’ by stronger repulsion force?).*

To address the first part of this question, we have varied the size of the perturbations we added to the division angle orientation in Figure 6 of the revised manuscript. Informed by the reported

angle distributions in Kaucke et al, we considered a maximal deviation of 12.5° from strictly vertical cell division orientations. For this maximal value, columnar structures are still present, although our order parameter, the envelope projection area, is larger than experimentally observed values in this case.

To address the second part of this question: Increasing the strength of the repulsive force would not straighten columns in our simulations. The force law in equation (1) of the revised manuscript determines the timescale of cell movement by the fraction η/μ, where η is the friction coefficient and μ is the strength of the repulsive force. Changing the strength parameter μ would thus simply change the speed at which cells move during mechanical interactions, and movement paths in rescaled coordinates would be unaffected, provided that movement remains in the adiabatic limit in which the timescale of mechanical rearrangement is much shorter than the timescale of cell division.

f. *Does the strength of activation by BMP influence how much cell divisions are aligned with the transversal direction? Can this be investigated using this model?*

Yes, the strength of BMP activation can influence how much cell divisions are aligned with the transversal direction. In experiments by Kaucka et al (2017), constitutive overactivation of ACVR1, a receptor of BMP on the cell surface, led to misoriented cell division, as the overactivation prevented read-out of the BMP gradient. While our simulations allow us to change division directions on a rule-based basis, as we have done in Figures 5 and 6 of the revised manuscript, it is outside the scope of the study to simulate components of the BMP pathway inside individual cells.

2. *The current model assumes that the influence of the ECM (extracellular matrix) on chondrocyte behaviour can be implicitly captured by setting an appropriate friction coefficient. However, this approach may not be sufficient in three-dimensional environments where cells are embedded in a fibrous ECM mesh with mechanical properties. In such scenarios, it is unclear how chondrocytes invade the ECM and whether they can degrade the surrounding matrix through proteolysis.*
   The authors suggest that when ECM is present in the gaps between cells, monoclonal columns are more transversally oriented. However, this conclusion seems questionable given the pre-imposed honeycomb pattern and the increased spacing between cell centres at t = 0, which decrease cell-to-cell mechanical interactions and would be expected to result in vertically oriented columns. Furthermore, the numerical experiment of column insertion in place of a deleted column yields predictable results that provide little insight. Additionally, if such large spaces filled with ECM were available for column insertions, they would likely be visible in experimental results (e.g. Figures from Kaucka et al. [1]).

We agree that there may be biological complexity that is not captured by our model, such as the 3D structure of the ECM or proteolysis. Simplification is an integral part of any modelling approach. A more accurate representation of the ECM may be part of future modelling studies, as we discussed in the original manuscript (page 32, lines 719ff of the revised manuscript).

Our simulation of column insertion demonstrates in a straight-forward way that increased available space through deposited ECM may enable column insertion, which we could not achieve in previous experiments of the paper. This is a new insight that can be tested experimentally.

We agree with the reviewer that it is unrealistic to assume that space for a whole column is pre-formed in this way by the ECM. Instead, cells could secrete ECM in the vertical direction before cell division, allowing for space to become available one cell at a time. To address this comment in the revised manuscript, we added this latter point to the discussion on page 32, line 704.

Minor issues:

3. *On line 119, "not sufficient" is perhaps too strong since no sensitivity analysis or model calibration was presented. The authors might want to soften this sentence.*

We addressed this comment by adding 'in our simulations' to the sentence.

4. *Can the authors clarify somewhere within the manuscript how the second (boundary) perichondrial cell appears? Or both perichondrial cells are present at cartilage sheet boundaries, and both divide?*

We apologise for this confusion, we should not have named the second cell a perichondrial cell and called it a chondrocyte instead. This is corrected in the revised manuscript on page 27, line 584. How this first chondrocyte cell appears is described on lines 583ff of the revised manuscript.

5. *Can the authors explain their choice of the initial honeycomb arrangement of cells in the x-y plane? This setup makes sense for the first numerical scenario where the authors test whether random division orientation combined with regular cell positioning is sufficient to generate monoclonal columns. However, for later numerical experiments, this might be an unnecessary restriction (even if slightly perturbed). Are there some experimental data available for mesenchymal condensations?*

Unfortunately, no experimental data are available on the lateral distribution of progenitor cells in mesenchymal condensations. Kaucka et al measured the thickness of the mesenchymal condensations and reported that these are only 1-2 cell diameters in height (video 1 of Kaucka et al. (2020)). This motivated us to consider initial mesenchymal condensations with only one cell in the z-direction. We consider the current setup of a hexagonal initial mesenchymal condensation in the lateral direction beneficial, as it allows a high average cell density in the x-y direction of the simulation. Comparable densities would be difficult to achieve with randomly chosen initial conditions, say. We addressed this comment by adding a sentence about this to the discussion on page 30, line 667.

6. *On line 407 and also in Figure 6 and its caption, the coordinate of the midsection should be $z = u+l$ instead of $z = u-l$ (although, I understand that since $l = 0$, this is not going to change simulation results).*

We thank the reviewer for noticing this typo. We have fixed this in the revised manuscript.

7. *I would like to suggest using the same text in (the beginning of) captions of Figures 2 and 3 (illustrating the two initial conditions used in simulations) as in the titles of the*

subsections, which introduce the corresponding initial condition (and refer to these initial conditions in a similar way, e.g. Configuration (i)).

This is a helpful suggestion, which we have implemented in the revised manuscript.

8. *Around line 420, could the authors clarify whether for the numerical experiments of tissue thickening u is adjusted at the time, t = 0, or it is gradually increased during simulations?*

This adjustment is made in the initial condition, at time t=0. We have clarified this in the revised manuscript on page 23, line 528 of the revised manuscript.

9. *Can stronger repulsion force contribute to column straightening (in general and in experiments of tissue thickening and lateral expansion by column insertion)?*

No, stronger repulsive forces cannot contribute to column straightening in our model. Increasing the strength $\boldsymbol{\mu}$ of the repulsive force will change the timescale of cell rearrangement, but not the paths the cell centres take during the rearrangement. This can be seen in equation (1) of the revised manuscript, where the characteristic time scale of the ODE is set by $\boldsymbol{\eta}/\boldsymbol{\mu}$. This dependency was mentioned in the original manuscript (page 12, line 272 of the revised manuscript).

10. *Could the authors clarify the sentence on lines 227-228?*

We have clarified this sentence on line 243 of the revised manuscript.

11. *On line 473, it is indicated that 10 cells were activated to divide for the column insertion experiment. However, I see only 9 activated cells in Figure 9.*

We thank the reviewer for noticing this typo. It has been fixed in the revised manuscript.

12. *On line 244, the authors state that perichondrial cells divide asymmetrically, but on lines 475-476, the division of perichondrial cells in the numerical experiment with column insertions is symmetrical. Is the division of perichondrial cells context-dependent? Could the authors clarify this within the text?*

We apologise that this was confusing. We should not have named the second cell a perichondrial cell and called it a chondrocyte instead. This is corrected in the revised manuscript on page 27, line 584.

13. *Could the authors add a reference supporting their statement that "cell adhesion molecules are not present" in chondrocytes (lines 158-159)? (Is it the same work cited in the following sentence?)*

We thank the reviewer for noticing this. It is not the same reference as the next sentence. We have added the correct reference (Oberlender et al, 1994) to address this.

14. *In the code of the model shared by the authors on GitHub, I would like to suggest that the project name cartilage in all CMakeLists.txt files (chaste do apps projects(cartilage)) is changed to CartilageCBM as the name of the GitHub repository (otherwise, users will have to do it themselves to be able to compile and run the code).*

This is a helpful suggestion. We have made this change in our code.
